# Evaluation of antioxidant potential and reduction capacity of some plant extracts in silver nanoparticles' synthesis

**Published:** 2014-09

**Authors:** Vahid Goodarzi, Hajar Zamani, Leila Bajuli, Ali Moradshahi

**Affiliations:** 1 Department of Biology, Faculty of Sciences, Shiraz University, Shiraz 71454, Iran

**Keywords:** Antioxidant potential, Reduction capacity, DPPH, Folin-Ciocalteu, Silver nanoparticles

## Abstract

The green synthesis of metallic nanoparticles is an active research area in nanotechnology. In the present study, antioxidant potential, total reducing capacity and silver nanoparticles' (Ag NPs) synthetic potential of methanolic leaf extracts of seven plant species were evaluated and compared. Antioxidant capacity, expressed as µmol Trolox equivalents g^-1^ DW (µmol TE g^-1^ DW), ranged from 116.0 to 1.80. The plants *Rosmarinus* sp. and *Zataria Multiflora *showed highest antioxidant capacities with IC_50_ of 1.07 and 1.22 mg ml^-1^, respectively. Total reducing capacity ranged from 7.6 to 0.17 mg gallic acid equivalent to g^-1^ DW (mg GAE g^-1^ DW). Plants with high antioxidant potentials also showed higher total reducing capacity. In fact, the order of the plants' reducing capacity was similar to that of their antioxidant potential. The same two plant species, i.e., *Zataria Multiflora* and *Rosmarinus* sp, with high reducing capacities, showed higher potentials for Ag NPs synthesis. It is concluded that reducing substances in the extracts contribute significantly to the antioxidant potential of the tested plant species, and plants with a high reducing capacity are excellent sources for the green synthesis of metallic nanoparticles. In addition, synthetic antioxidants have adverse effects on human health; therefore, to benefit more from the health promoting properties of plant species, evaluating their novel natural antioxidants is recommended.

## INTRODUCTION

Reactive oxygen species (ROS) are produced during normal cellular metabolism such as respiration and photosynthesis and are scavenged by antioxidant defense systems of the organisms [1-7]. Under different stress conditions, the rate of ROS production overrides the rate of ROS removal, leading to oxidative stress in the organisms [1,8-11].Oxidative stress generated by ROS damages macromolecules such as proteins, lipids and nucleic acids and often leads to metabolic dysfunction and cell death [6,12-14].

Due to their beneficial health effects, the use of antioxidants as food supplements or ingredients has recently increased. Since synthetic antioxidants may have negative health effects, many plant species are investigated for novel natural antioxidants [15-19]. Commercially available natural antioxidants are often derived from terrestrial plants. Many plants contain large amounts of antioxidants such as phenolic compounds, vitamin E, vitamin C and carotenoids [20-24]. 

The estimation of antioxidant potential and total reducing capacity of living organisms such as algae and plants is carried out by different assay methods [25-28]. These methods are classified as electron and hydrogen-atom transfer-based assays. DPPH, FRAP and Folin-Ciocalteu assays are examples of electron transfer-based assays [29,30]. In these redox reactions, the antioxidants present in the sample transfer electrons to oxidants such as DPPH radical or to the metal ion present in Folin-Ciocalteu reagent. Therefore, by recording changes in absorbance at a specific wavelength, the total reducing capacity of a sample can be calculated from a standard curve. 

Redox reaction also occurs during metallic nanoparticle synthesis from corresponding aqueous salts. Extracts from different organisms contain reducing substances which transfer electrons to the metal ions producing metallic nanoparticles [31-33]. Plants with higher total reducing substances should be able to produce higher concentrations of metallic nanoparticles. One novel Ag NPs-based assay has been proposed by Szydlowska-Czerniak et al. [6] to determine total reducing capacity. Correlations between the suggested assay with electron transfer-based assays such as DPPH and F-C assays, were shown to be significant (0.76 – 0.85, *P˂0.05*). Moreover, the plots of absorbance at 410 versus concentrations of standard phenolic compounds were found to be linear.

In the present study antioxidant potential and total reducing capacity of methanolic leaf extracts of seven plant species were determined and compared. The hypothesis that extracts with higher total reducing capacity are more potent in green synthesis of Ag NPs was evaluated and plants with high Ag NPs synthetic potential were identified.

## MATERIALS AND METHODS

**Reagents and extraction procedure:**All reagents were of analytical grade and obtained from Sigma-Aldrich Chemical Co. (Darmstadt, Germany). 

Aerial parts of collected plants were washed and dried at room temperature in the dark. One gram of powdered leaves of each plant was extracted with 30 ml methanol for one day at room temperature in the dark. After centrifugation at 4000 rpm for 10 min, the supernatants were used to determine antioxidant and total reducing capacity. For green synthesis of Ag NPs, one gram of each powdered leaf in 30 ml deionized water was boiled for 5 min. After centrifugation at 4000 rpm for 15 min, the supernatant was diluted with deionized water to give extract concentrations corresponding to 1.0 to 30 mg ml^-1^ of the original plant material to be used for the Ag NPs synthesis.

**Measurement of radical scavenging capacity by DPPH assay: **DPPH assay was performed as described by Thaipong et al. [34]. In brief, 150 µl standard solution or sample was added to 2850 µL DPPH solution and kept in the dark. After 60 min, the absorbance of the solution was measured at 515 nm. Trolox, in the concentration range of 25 to 800 nmol in methanol, was used to construct the calibration curve. Radical scavenging is reported as µmol trolox equivalent per gram dry weight (µmol TE g^-1^ DW). The percent inhibition of the DPPH radical by one ml of the extracts was calculated according to the following equation_˸_

% inhibition = [(A_c _-A_s_) _/_ A_c_] _×_100,

where A_c_ is the control absorbance at 515 nm and A_s_ is the sample absorbance after 60 min of incubation. 

The methanolic extract concentration that scavenged 50% of the DPPH radicals (IC_50_) was calculated from the DPPH absorbance plot at 515 nm versus the extract concentration.

**Determination of total reducing capacity: **Folin-Ciocalteu (F-C) assay was performed as described by Velioglu et al. [21]. In brief, 200 µl standard solution or sample was mixed with 1.5 mL of Folin-Ciocalteu reagent previously diluted tenfold with distilled water. After 5 min, 1.5 ml of 6% (w/v) sodium bicarbonate solution was added to the solution. The mixture was kept for 90 min at room temperature and the absorbance was reordered at 750 nm. Gallic acid was used as the standard in the concentration range of 25-200 µg ml^-1^. 

**Green synthesis of silver nanoparticles: **For green synthesis of Ag NPs, 100 µl of each sample was added to and mixed thoroughly with 2.0 ml of 1.0 mM AgNO_3_ at room temperature. Synthesis of Ag NPs after 3 hrs of incubation was determined by recording absorbance at 410 nm using a double beam spectrophotometer (SHIMADZU160A). The reaction was carried out at ambient temperature and pH=7.0.

**Statistical analysis:**Each sample had three replicates and data )shown as mean ± SE and means( were compared using SPSS 16.0. Duncan^’^s multiple range test was used to determine significant differences at *p < 0.05*. Correlation analysis was carried out using Pearson’s correlation and regression analysis using SPSS version 16.0.

## RESULTS AND DISCUSSION

**Figure 1:**
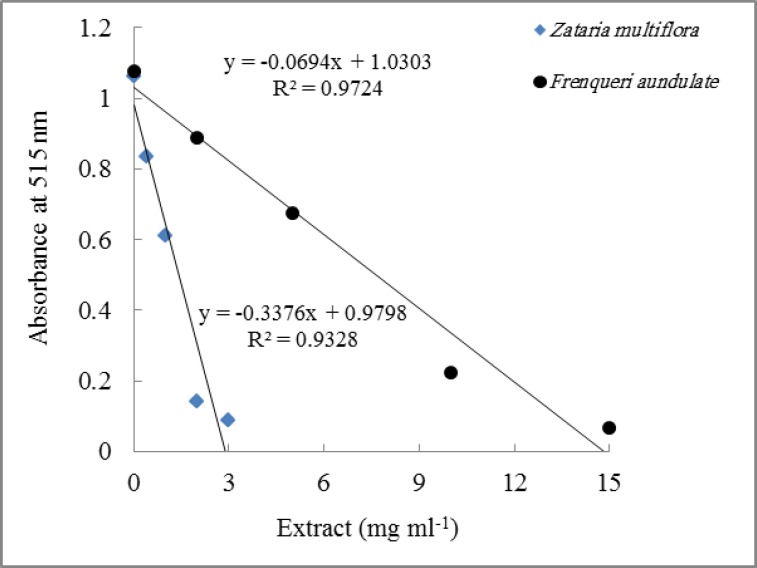
Effects of different concentrations of methanolic leaf extracts of* Zataria multiflora* and *Francoeuria undulate* on DPPH absorbance at 515 nm

**Antioxidant capacity:** Table 1 shows antioxidant capacity together with percent inhibition of DPPH radicals and IC_50_ of the methanolic leaf extracts of seven plant species. Antioxidant capacity ranged from 116.0 to 1.8 µmol TE g^-1^ DW with *Rosmarinus* sp. and *Zataria Multiflora* having the highest antioxidant capacity. Percent inhibition of DPPH radicals followed the same order as antioxidant capacity and ranged from 93.4 to 3.03 percent. IC_50_ of 1.07 to 25.20 mg ml^-1^ was obtained for tested extracts which were inversely related to antioxidant capacity. The plots of absorbance of DPPH radicals at 515 nm versus extract concentrations were linear for all the extracts with R^2^˃0.98 shown for the plants *Zataria Multiflora* and *Francoeuria undulate* (Fig. 1). For *Zataria Multiflora,* about 3.0 mg ml^-1^ was needed to reduce absorbance from 1.06 to 0.09, whereas for *Francoeuria undulate,* about 15 mg ml^-1^ was required for the same reduction in absorbance at 515 nm.

**Table 1:** Antioxidant capacity, percent DPPH inhibition and IC_50_ of leaf extracts of seven plant species. Data are mean ± SE. In each column values with different letters are significant at *P<0.05*.

Plant material	Trolox equivalents mol g^-1^ DW)µ)	% Inhibition of DPPH	IC_50 _ ( mg ml^-1 ^)
Rosmarinus sp.	116.00±0.001^a^	93.40±0.17%^a^	1.07±0.01^a^
Zataria multiflora	108.00±0.008^b^	86.65±0.85%^b^	1.22±0.04^a^
Pelargonium graveolens	36.00±0.007^c^	27.88±2.18%^c^	3.56±0.08^b^
Chamaemelum nobil	7.60±0.006^d^	20.65±0.31%^c^	4.74±0.04^c^
Frenqueri aundulate	4.20±0.001^e^	17.56±0.11%^d^	6.86±0.11^d^
Achillea wilhemsii	3.00±0.009^f^	11.49±0.46%^e^	7.61±0.16^e^
Carthamus tinctorius L.	1.80±0.005^g^	3.03±0.45%^f^	25.20±0.24^f^

**Figure 2:**
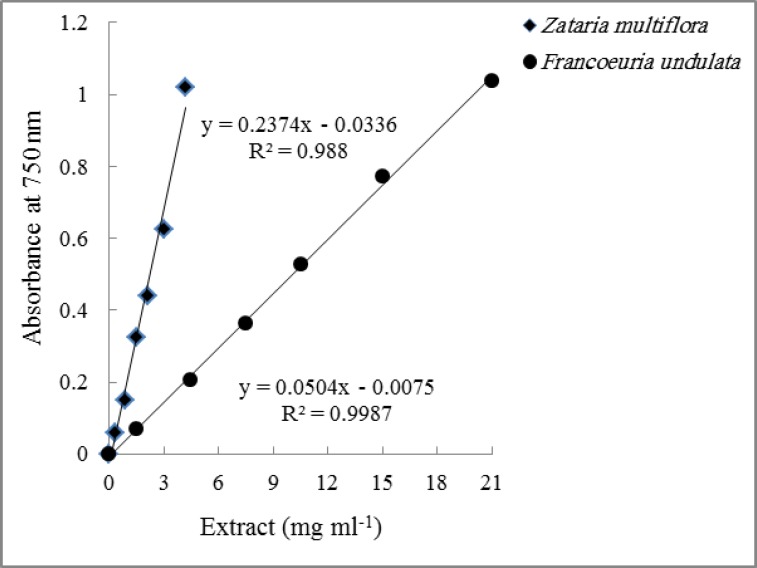
Effects of different extract concentrations of *Zataria multiflora* and *Francoeuria undulate* on Folin-Ciocalteu reagent at 750 nm

**Total reducing capacity: **The results of the Folin-Ciocalteu assay which measures total reducing capacity and also gives an estimate of total phenolics content are shown in Table 2. The order of total reducing capacity was very similar to that of antioxidant capacity indicating close relationships between these two parameters in the tested plant species. Total reducing capacity ranged from 7.60 to 0.17 mg GAE g^-1^ DW. The plots of extract concentrations versus absorbance at 750 nm were linear for all extracts shown for *Zataria Multiflora *and *Francoeuria undulate* in Figure 2. For *Zataria Multiflora,* about 3 mg ml^-1^ was required to increase absorbance at 750 from zero to about 1.0, whereas for *Francoeuria undulate*, about 21 mg ml^-1^ of extract was required to get the same increase in absorbance. 

**Table 2:** Total reducing capacity and AgNPs synthetic potential of methanolic leaf extracts of seven plant species. Each value is mean± SE. Values with different letters are significantly different at *P < 0.05*. AgNPs production by 2 mg ml^-1^ of the extracts was compared after 3 hrs of incubation.

Plant material	Total reducing capacity (mg GAE g^-1^ DW)	AgNPs synthesis (absorbance at 410 nm)
*Zataria multiflora*	7.60±0.33^a^	0.92±0.17^a^
*Rosmarinus *sp*.*	6.00±0.29^b^	1.07±0.16^a^
*Pelargonium graveolens*	2.96±0.11^c^	0.24±0.07^b^
*Chamaemelum nobil*	0.56±0.16^d^	0.21±0.025^b^
*Frenqueri aundulate*	0.46±0.17^d^	0.40±0.022^b^
*Achillea wilhemsii*	0.42±0.00^d^	0.36±0.20^c^
*Carthamus tinctorius L.*	0.17±0.05^e^	0.23±0.08^b^

**Figure 3:**
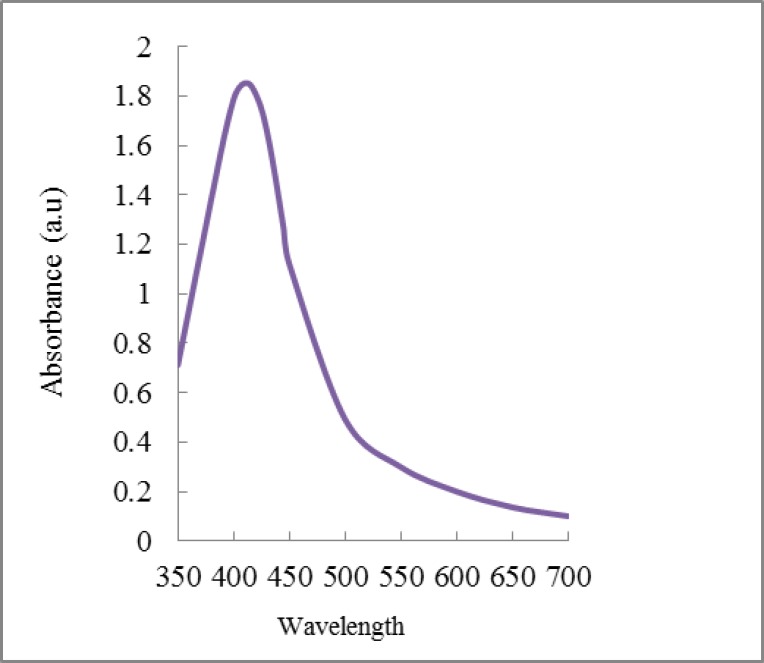
UV-Vis spectrum of silver nanoparticles synthesized due to the reduction of silver ions by the leaf extracts

**Silver nanoparticles synthesis: **Synthesis of Ag NPs with 2 mg ml^-1^ of the leaf extracts was determined at 410 nm which is the wavelength of maximum absorption of Ag NPs (Fig. 3). As shown in Table 2, based on the Ag NPs synthetic potential, the plants could be divided into two groups. *Zataria Multiflora* and *Rosmarinus* sp., with high total reducing capacity, were more potent in Ag NPs synthesis, whereas plants with lower total reducing capacity showed less potential for the synthesis of Ag NPs.

Developing high-yield, low cost, nontoxic and environmentally friendly methods for metallic nanoparticles synthesis [31] is an increasing need. Reduction of silver ions to Ag NPs by plant extracts have been reported by several investigators [35-41]. Stable Ag NPs in the size range of 16 to 40 nm has been reported using geranium leaf extracts. Terpenoids in the extract contributed to the reduction of silver ions to Ag NPs [35]. Using five plant leaf extracts, Song and Kim [33] reported a rapid green synthesis of Ag NPs. Magnolia leaf extract was the best reducing agent in terms of synthetic rate. Using Neem (*Azadirachta indica*) leaf broth, Shankar et al. [42] showed the synthesis of pure metallic nanoparticles of silver and gold. Green synthesis of Ag NPs has also been reported using curry leaf (*Murraya koenigii*) extract [32]. Switch grass (*Panicum virgutum*) extract mediated the green synthesis of Ag NPs from a silver nitrate solution at ambient temperature [43]. Since antioxidant potential and total reducing capacity vary among different plant species [44-46], it is expected that plants with higher reducing capacity are more potent in reducing metallic ions to metallic nanoparticles. In the present study, plant extracts with higher antioxidant potentials and higher reducing capacity caused higher absorbance at 410 nm, which is indicative of a higher level of Ag NPs production. Phenolic compounds are the major constituents of antioxidants of most plant species and their antioxidant activity is mainly due to their redox properties. As a result, they can act as reducing agents in neutralizing free radicals [47,48] and the reduction of metallic ions to metallic nanoparticles. Similar to the present study, Schwarz et al. [28] showed that rosemary extract with high phenolic compounds was one of the best antioxidant sources among the plant extracts tested. This plant can therefore, be used efficiently as a reducing agent in the synthesis of metallic nanoparticles. 

Plants with high antioxidant and reducing capacities are not only useful for the green synthesis of metallic NPs, but also for the prevention or reduction of the harmful effects of reactive oxygen species (ROS) generated during normal cellular metabolism of plants and animals [1,7]. Natural antioxidants such as rosemary, sage and zataria are already used commercially as antioxidant additives or nutritional supplements [49]. In the present study, the two plants *Rosmarinus* sp. and *Zataria Multiflora* showed the highest antioxidant and reducing capacities. More plant species need to be evaluated for their novel antioxidants and reducing substances; first, to benefit from their potential health advantages including protection against disorders such as cancer and cardiovascular diseases and next, to synthesize green and eco-friendly metallic nanoparticles. 
